# Application of Telemedicine in the Management of Cardiovascular Diseases: A Focus on Heart Failure

**DOI:** 10.31083/RCM37835

**Published:** 2025-07-01

**Authors:** Pornthira Mutirangura, Dil Patel, Hassan Akram, Andrew Hughes, Jose Arriola-Montenegro, Despoina Koukousaki, Joel Money, Marinos Kosmopoulos, Mikhail Meyer, Mikako Harata, Andras Toth, Tamas Alexy

**Affiliations:** ^1^Department of Medicine, University of Minnesota, Minneapolis, MN 55455, USA; ^2^Department of Medicine, Division of Cardiology, University of Minnesota, Minneapolis, MN 55455, USA; ^3^Department of Medicine, Division of Nephrology, Mayo Clinic, Rochester, MN 55905, USA; ^4^University of Minnesota, Minneapolis, MN 55455, USA; ^5^Department of Medical Imaging, University of Pecs, 7622 Pecs, Hungary

**Keywords:** heart failure, telemedicine, remote heart failure management, noninvasive monitoring

## Abstract

Heart failure (HF) is a complex clinical syndrome that represents one of the leading causes of morbidity and mortality in developed nations. It is well established that every HF-related hospital admission leads to worsened quality of life for the patient and their caregiver and also imposes a significant financial burden on society. Therefore, reducing hospital admissions for this population has emerged as a critical tactic over the past decades. Initial attempts at remote monitoring focused on self-reported vital signs and symptoms, yet these proved ineffective. Meanwhile, subsequent technological advancements have enabled the development of miniature sensors capable of detecting and monitoring a wide range of physiologically relevant parameters; some of these advancements have been integrated into implantable devices, such as pacemakers and defibrillators. However, noninvasive monitoring has recently emerged as an alternative option for patients with HF, offering early congestion detection without requiring an invasive procedure. This review aims to summarize implanted and noninvasive devices, their characteristics, monitored parameters, and potential limitations and challenges around their integration into routine clinical practices.

## 1. Introduction

Heart failure (HF) is estimated to affect over 64 million people worldwide, with continually rising incidence and prevalence rates [1]. Despite the significant effort invested in developing and introducing novel medical and device-based therapies, HF-related hospitalizations remain unacceptably frequent, contributing to the high mortality in this population as well as the reduced quality of life [2]. It has also been established that inpatient admissions are responsible for most management-associated expenditures, representing a significant financial burden for society [3, 4]. Early detection of worsening HF, increasing congestion in most cases, is essential to improve outcomes. However, achieving this goal has proven challenging owing primarily to the fact that the common signs and symptoms of fluid retention that patients experience, such as weight gain, lower extremity edema, dyspnea, orthopnea, and early satiety, only manifest late and seeking medical attention is typically unavoidable at this stage [5, 6, 7]. Thus, the idea of telemedicine was conceived in response to this obstacle. However, given the technological limitations, the initial remote HF monitoring focused on telephone calls initiated by the patient or the care team either at set periods or when changes in weight, symptoms, or select vital signs occurred. Despite the initial promising findings in small studies, larger, randomized, controlled trials failed to demonstrate a consistent benefit for such interventions in reducing HF-related rehospitalizations and all-cause mortality [8, 9, 10, 11]. Close examination of these trials revealed that near real-time remote management was associated with better outcomes, yet the ideal parameters for monitoring remained undefined.

It was soon recognized that a rise in intracardiac filling pressures represented the earliest detectable evidence of HF decompensation [12, 13, 14, 15]. Indeed, increased intracardiac filling pressures may start several weeks before actual symptom development due to progressive neurohormonal activation to maintain homeostasis [12]. Therefore, this period may represent a window of opportunity for interventions to slow or stop the path to decompensation effectively; however, translating this observation into routine clinical practice has proven challenging. While right heart catheterization (RHC) remains the gold standard for detecting congestion [16], this method is an invasive procedure that is not universally available, typically requires a hospital setting, and is not feasible to perform repeatedly to monitor day-to-day hemodynamic changes. Consequently, there has been a shift towards developing implantable devices that facilitate the remote daily evaluation of filling pressures, at the convenience of the patient’s residence [15, 17, 18, 19, 20, 21]. Recent advancements in technology have enabled the design of miniature sensors capable of monitoring a wide range of relevant physiological variables in addition to intravascular pressure. Given the frequent use of implantable cardioverter-defibrillators (ICDs) and cardiac resynchronization therapy-defibrillators (CRT-Ds) in the HF population [22], several of these sensors were successfully integrated into these devices [23, 24, 25]. In addition, the development of noninvasive monitoring technologies has accelerated recently, with several undergoing active testing as potential alternatives to those requiring implantation. Thus, utilizing these would eliminate all risks associated with the procedure, the need for possible future exchanges, infections, and the mandated use of antiplatelet agents in many instances [26, 27].

​​This review aimed to briefly overview selected invasive and emerging noninvasive technologies that enable early, remote detection of worsening congestion and facilitate appropriate interventions. These can potentially prevent hospital admissions and improve the quantity/quality of life of the population living with HF.

## 2. Methods

To complete this manuscript, PubMed, ScienceDirect, and other online scientific databases were searched and reviewed to identify devices aiming to detect HF exacerbation. Keywords used for the initial searches included “heart failure”, “outpatient”, “heart failure devices”, “heart failure monitoring”, “remote heart failure management”, and “telemedicine”. Subsequent searches were narrowed and included the specific devices that were identified earlier. Here, we only considered relevant peer-reviewed articles, completed/ongoing clinical trials, and expert opinions published within the past 20 years. Information regarding device types, stage of development, commercial availability, monitored parameters, reported clinical outcomes, and potential complications was extracted for each platform.

## 3. Literature Review

### 3.1 Implantable Remote Hemodynamic Monitoring Platforms

Once it became evident that elevated intracardiac filling pressures are the primary driver for HF symptoms, a variety of implantable hemodynamic monitoring platforms. A summary of these devices is provided in (Table 1, Ref. [13, 17, 18, 20, 21, 28, 29, 30]) with a more detailed description of each below.

**Table 1.** **Selected implantable remote hemodynamic monitoring platforms**.

Device	Monitored parameters	Indications/population studied	Contraindications	Relevant trials
CardioMEMs^TM^	PA pressure	FDA approved:	● Inability to administer DAPT or AC for one month post-implantation	CHAMPION [13]
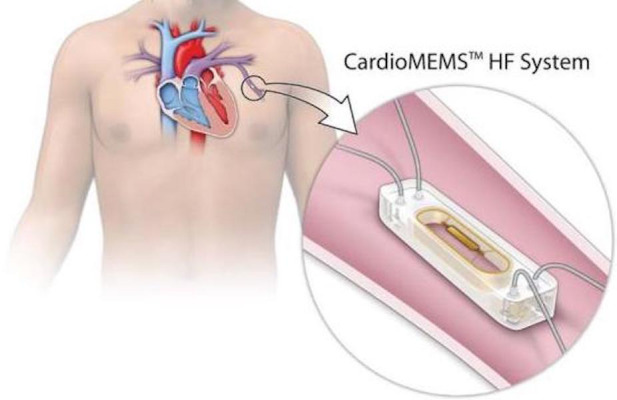		● NYHA Class II–III symptoms and		GUIDE-HF [17]
		● At least one HF hospitalization within the preceding 12 months and/or		MONITOR-HF [30]
		● Elevated natriuretic peptides		
Cordella^TM^ heart failure system	PA pressure, vital signs	FDA approved:	● Inability to administer DAPT or AC for one month post-implantation	SIRONA [28]
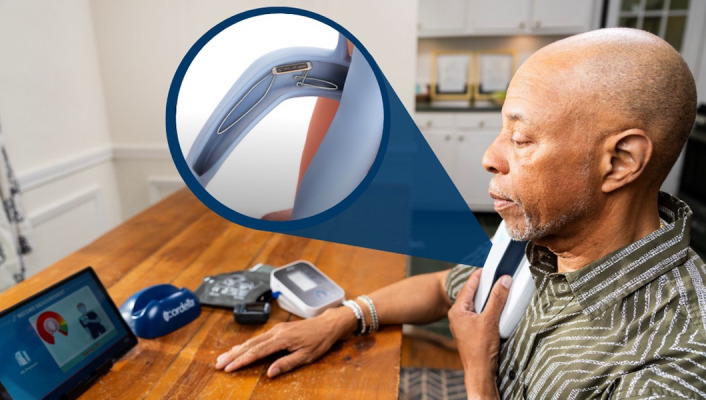		● NYHA Class III symptoms and		SIRONA II [29]
		● At least one HF hospitalization within the preceding 12 months and/or		PROACTIVE-HF [18]
		● Elevated natriuretic peptide levels		
V-LAP	LA pressure	Studied in:	● Inappropriate left atrial and interatrial septal anatomy	VECTOR-HF [20]
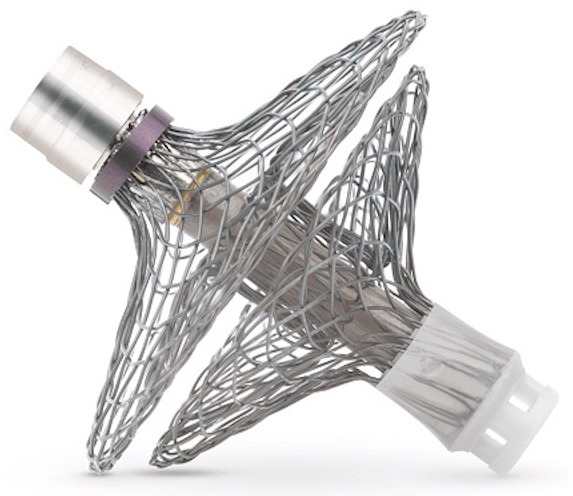		● NYHA Class III symptoms and	● Atrial septal defect or patent foramen ovale with more than a trace amount of shunting	
		● At least one HF hospitalization in the preceding 12 months and/or		
			● Inability to administer DAPT or AC for three months post-implantation	
		● Elevated natriuretic peptide levels		
FIRE-1	IVC pressure	Studied in:	● Inability to administer DAPT or AC for one month post-implant and SAPT or AC throughout life	FUTURE-HF
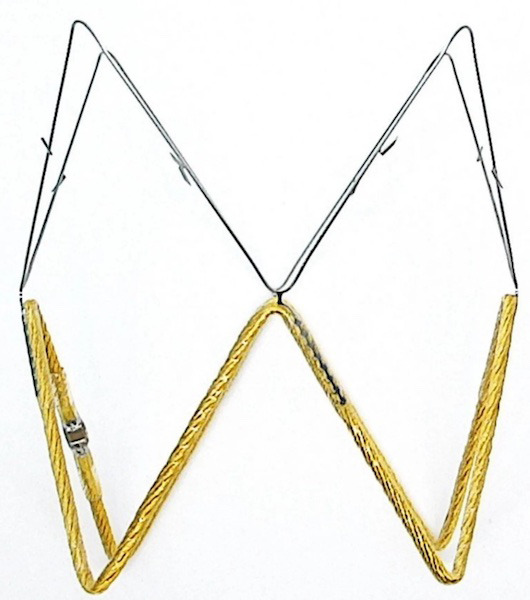		● Any NYHA Class and		(NCT04203576)
		● At least one HF hospitalization in the preceding 12 months and		FUTURE-HF2 [21]
		● Elevated natriuretic peptides and		
		● Patient taking ≥40 mg daily furosemide equivalent		

AC, anticoagulation; DAPT, dual antiplatelet therapy; FDA, Food and Drug Administration; HF, heart failure; IVC, inferior vena cava; LA, left atrium; NYHA, New York Heart Association; PA, pulmonary artery; SAPT, single antiplatelet therapy.

#### 3.1.1 HeartPOD and V-LAP Left Atrial Pressure Monitoring Devices

As it became increasingly evident that remote vital sign monitoring alone provides limited benefits in improving HF outcomes, results from animal models suggested a strong correlation between left atrial (LA) pressure (a direct reflection of left ventricular end diastolic pressure) and pulmonary congestion [31, 32]. Based on these observations, the implantable HeartPOD system (Abbott Laboratories, Minneapolis, MN, USA) was developed to measure LA pressure directly. The platform resembled a transvenous pacemaker consisting of two main components: (1) a pressure sensor embedded into the tip of a lead threaded across the interatrial septum; (2) a subcutaneous antenna coil enabling wireless communication with the patient advisory module. The collected data were automatically sent for review by the clinical team responsible for monitoring and interventions. HOMEOSTASIS was the first-in-human study that enrolled 40 patients with New York Heart Association (NYHA) Class III–IV symptoms and showed improved hemodynamics, symptom burden, and outcomes with physician-directed patient self-management [33]. The subsequent randomized, controlled, multicenter LAPTOP-HF trial was stopped early, however, due to the excess of implant-related complications, which included aortic puncture, cardiac free-wall perforation, disruption of the conduction system, in addition to other major adverse cardiovascular and neurological events [19]. The trial was designed to enroll 730 participants, but it stopped early after 486 patients exhibited an unacceptably high procedure-related complication rate. Overall, a negative study failed to demonstrate a significant reduction in the combined endpoint of recurrent HF hospitalizations and complications of HF therapy [19]. However, it stimulated further device development and paved the way for the second generation of LA pressure sensors, such as the V-LAP.

V-LAP (Vectorious Medical Technologies, Tel Aviv, Israel) represents a recently developed, Food and Drug Administration (FDA)-approved remote LA pressure monitoring platform for patients with chronic HF. The system consists of two individual units: (1) a miniature, leadless, hermetically sealed pressure sensor that traverses the interatrial septum and is held in a stable position by two nitinol discs deployed on either side of the septum; (2) a portable crossbody belt. The sensor is implanted during an RHC procedure combined with transseptal puncture. The belt communicates wirelessly with the sensor to receive real-time data while supplying the necessary power. The belt then uploads the received information directly to a secure, cloud-based platform easily accessible to patients and their provider team. Importantly, the system is set up to utilize a “manage-by-exception” strategy, which will send an automated notification to responsible providers should a significant rise in LA pressure occur. As such, this change may represent the first hemodynamic sign of impending HF exacerbation; thus, immediate attention and intervention will likely prevent progression to symptomatic congestion necessitating hospital admission or emergency room (ER) visit for diuresis. The safety, usability, and performance of this technology were assessed in the first-in-human, prospective, single-arm, multicenter VECTOR-HF trial that enrolled 30 patients with NYHA Class III symptoms despite maximally tolerated guideline-directed medical therapy (GDMT), irrespective of left ventricular ejection fraction (LVEF) [20]. The study by D’Amario *et al*. [20] revealed a very close correlation between the LA pressure as measured by the V-LAP platform and the pulmonary capillary wedge pressure (PCWP) obtained during invasive hemodynamic evaluation (–0.22 ± 4.92 mmHg, r = 0.79; *p* < 0.0001). V-LAP-guided HF management led to a significant improvement in self-reported NYHA functional class: 32% of participants reached Class II symptoms by 6 months (*p* < 0.005) and 60% by one year (*p* < 0.005) [20]. In addition, there was a significant improvement in 6-minute walking distance (*p* < 0.005); complications related to the implant procedure may occur, but none were observed during the clinical trial. Furthermore, long-term complications may include an increased risk for arrhythmias, thrombus development over the nitinol frame, and infection. Freedom from major adverse cardiac and neurological events was 97% at 3 months. While the safety profile appears excellent, larger trials and long-term real-world data are necessary to ascertain this success. Overall, the limited evidence suggests a strong future potential for the V-LAP platform in the proactive management of patients with HF.

#### 3.1.2 Implantable Pulmonary Artery Pressure Sensor Systems

Given the potentially favorable association between direct LA pressure-based volume management and HF outcomes, research turned towards developing novel hemodynamic pressure sensors that did not require septal puncture. It became evident that, for most patients, there is a relatively close correlation between the diastolic pulmonary artery (PAD) pressure and PCWP, with the latter closely related to LA pressure. This led to the development of miniature, implantable pulmonary artery (PA) pressure sensors.

#### 3.1.3 CardioMEMs^TM^

CardioMEMs^TM^ (Abbott Laboratories, Minneapolis, MN, USA) was the first FDA-approved implantable PA pressure sensor. This microsensor, deployed through a same-day procedure preferably to a branch PA measuring 7–10 mm on the left side, enables remote PA pressure monitoring from the homes of each patient. Daily readings acquired under one minute while lying supine on a specifically designed pillow were submitted automatically to a secure web platform (Merlin^TM^). Providers can review all readings or only those that trigger a warning by falling outside of the individually established target PA bracket. The care team then may choose to communicate with the patients at risk for decompensation and adjust their therapies promptly, thereby reducing the risk of symptomatic congestion and the need for medical attention.

CHAMPION was the first randomized, controlled trial focusing on CardioMEMs^TM^ and included 550 patients with NYHA Class III symptoms who had experienced at least one HF hospitalization within the prior 12 months [13]. The cohort, managed based on daily PA pressure readings, experienced a 28% reduction in HF-related hospitalizations at six months compared to the standard care group [13]. The most recently published multicenter GUIDE-HF prospective, randomized clinical trial expanded enrollment to those with NYHA Class II symptoms who required medical attention within the previous year for worsening HF symptoms or had elevated N-terminal pro-B-type natriuretic peptide (NT-proBNP) levels despite maximally tolerated GDMT [17]. A total of 1000 patients were enrolled over 21 months. While the coronavirus disease 2019 pandemic interfered profoundly with study execution, the pre-pandemic analysis performed with advanced agreement from the FDA showed a significant reduction in the composite endpoint of mortality and HF events (*p* = 0.049) [17]. Several independent, retrospective analyses have also been performed to analyze the potential benefits of the CardioMEMs^TM^ device. Desai *et al*. [34] demonstrated a 45% reduction in cumulative HF hospitalizations in the real-world setting, significantly reducing medical expenditures. The benefit occurred independently of the LVEF. The recently completed multicenter VICTOR trial (NCT05428384) was designed to expand the capabilities of the CardioMEMs^TM^ sensor by adding the ability to estimate cardiac output (CO) and cardiac index (CI). Knowing these variables could prove extremely beneficial, especially when caring for patients with more advanced disease onsets across the HF spectrum. Results are expected in the near future.

#### 3.1.4 Cordella^TM^ Heart Failure System

The Cordella^TM^ heart failure system (Edwards Life Sciences, Irvine, CA, USA) is another FDA-approved platform that consists of a pressure sensor implanted into the distal right PA, a central tablet that integrates all patient-related information, and a range of peripherals connected via Bluetooth for vital sign monitoring. These parameters include blood pressure, arterial oxygen saturation, and weight. The Cordella^TM^ PA sensor utilizes a proprietary delivery system and is deployed in the distal segment of the right PA [35]. Rather than lying flat on a pillow, daily readings are obtained in a supine position by placing a small handheld reader over the right anterior chest wall (although studies gathering data in a supine position are underway). The data are transmitted automatically to a secure web platform visible to the care team. Target PA pressure and vital sign ranges may be set individually for each patient, and customized notifications may be triggered when any variables fall outside this range. The SIRONA and SIRONA II trials completed in Europe established the safety of the device and confirmed an excellent concordance between the PA pressure obtained from invasive RHC and the implanted sensor [28, 29]. Following the recently published single-arm, multicenter PROACTIVE-HF trial, the Cordella^TM^ HF system received FDA approval for patients with NYHA Class III symptoms and elevated serum NT-proBNP levels or HF-related hospitalization within the preceding 12 months, independent of LVEF [18]. Notably, the study reported 0.15 HF-related hospitalizations or all-cause mortality events per patient (95% CI: 0.12–0.20) within the first six months, less than half the rate anticipated based on the MEMS-HF trial [18, 36].

Based on the emerging evidence, the 2022 American Heart Association/American College of Cardiology/Heart Failure Society of America guidelines for the management of HF, PA pressure sensors received a IIb recommendation for patients with NYHA Class III symptoms who required medical intervention for worsening HF within the preceding 12 months or have persistently elevated biomarkers despite maximally tolerated GDMT [22]. Studies aiming to strengthen the evidence are ongoing and are anticipated to expand the clinical indications for PA pressor sensors further in the future. However, it is important to emphasize that PA pressure sensor placement requires an invasive procedure using a large-bore venous sheath. This increases the risk of access-site bleeding, especially since dual antiplatelet therapy is mandatory for 1 month post-procedure, with aspirin continually administered throughout life. The rates for CardioMEMs^TM^ sensor migration are very low and zero for Cordella^TM^ owing to its unique design. Therefore, patients must maintain outstanding compliance with daily data acquisition to achieve maximal clinical benefit. One additional caveat to remember is that a gradient exceeding 3 mmHg between the PAD pressure and PCWP is not uncommon in patients with chronic HF and those with pulmonary hypertension, particularly Group 1. Hence, it is critical to carefully establish and document this gradient for each individual during sensor implantation to avoid over-diuresis solely based on PA pressure values [37]. Moreover, it is important to consider simultaneously obtained systemic blood pressure values when making management decisions, as hypertension will lead to elevated left ventricular end-diastolic pressure (LVEDP) and, consequently, PA pressures.

#### 3.1.5 FIRE1

The FIRE1 (Foundry Innovation and Research 1 Ltd, Dublin, Ireland) device is a novel sensor implanted via a minimally invasive percutaneous procedure into the inferior vena cava (IVC) and is designed to detect impending HF exacerbation early. The platform monitors changes in the cross-sectional area of the IVC in real-time, a variable thought to be more sensitive than atrial pressure in evaluating intravascular volume. Signals from the sensor are detected by a receiver belt (i.e., NORM belt) worn around the waist for a few minutes daily. Raw data are uploaded directly to a secure, cloud-based platform that utilizes a proprietary algorithm to convert these into clinically meaningful information representing volume status. This is accessible to patients through a mobile application and their providers, who can initiate clinical interventions based on the data. This system is undergoing clinical trials in the single-arm, multicenter FUTURE-HF (NCT04203576) and FUTURE-HF2 trials [21]. The sensor may be implanted with other devices that provide information on left-sided filling pressures, further improving clinical outcomes for patients with HF.

### 3.2 Heart Failure Monitoring Using Cardiovascular Implantable Electronic Devices (CIEDs)

Implantable ICD and CRT devices carry a Class I recommendation for a well-defined segment of patients with HF for primary or secondary prevention of sudden cardiac death. Therefore, in recognition, several CIEDs have been developed with an integrated feature to detect congestion or predict HF exacerbation by continually trending various physiological parameters. This remote monitoring feature may provide added value to the primary implant indication of ICDs and CRTs.

#### 3.2.1 OptiVol® Fluid Status Trend

OptiVol® fluid status trend (Medtronic Inc., Minneapolis, MN, USA) is available in select Medtronic-branded ICD and CRT pacemakers/defibrillators. This technology is based on monitoring thoracic impedance, a parameter correlating with intrathoracic volume status. The platform automatically alerts the care team when the thoracic impedance reaches a predetermined threshold value. The Mid HeFT feasibility study enrolled 33 patients who underwent device implantation with the OptiVol® feature [24]. During hospitalizations (25 events in 10 patients), there was a strong inverse correlation between thoracic impedance and PCWP (r = –0.61, *p* < 0.001) as well as net volume loss (r = –0.70, *p* < 0.001) [24]. A decrease in impedance preceded the development of clinical HF symptoms by an average of 15 days, suggesting that the OptiVol® parameter could offer an early warning of impending HF exacerbation [24]. While initial results were promising [38, 39], subsequent trials presented limitations in this technology. For example, Ypenburg *et al*. [40] found that only 33% of OptiVol® alerts were associated with clinical HF symptoms. ​​Yang *et al*. [41] reported that the positive predictive value (PPV) was only 20.4%; however, the rate of false positive alarms reached 79.6%, with similar results demonstrated in the prospective, multicenter SENSE-HF trial [42]. Finally, several subsequent randomized, controlled, multicenter studies failed to show a clinical benefit when utilizing OptiVol® alerts [43, 44, 45, 46]. Similarly, the CorVue system (St. Jude Medical, Sylmar, CA, USA) also had low accuracy in detecting HF decompensation episodes early [47, 48], ultimately limiting the use of these platforms in routine clinical practice.

#### 3.2.2 HeartLogic^TM^

The recently developed HeartLogic^TM^ (Boston Scientific, Marlborough, MA, USA) is an algorithm that integrates physiological data derived from various sensors in select ICD and CRT pacemakers/defibrillators to detect early signs of worsening congestion. These include cardiac sounds, thoracic impedance, respiratory patterns, body positioning, and routine physical activity. The care team is notified automatically when the HeartLogic^TM^ composite index rises above a predetermined threshold value, and adjustments in the HF management strategy may be initiated. The MultiSENSE non-randomized feasibility study enrolled 900 patients and demonstrated a 70% sensitivity in detecting HF events, defined as hospital admission or unscheduled visit requiring intravenous HF therapy [49]. The unexplained alert rate was 1.47 [1.32; 1.65] per patient–year [49]. The subsequent MANAGE-HF trial enrolled 200 patients with NYHA Class II–III HF symptoms, LVEF <35%, hospitalization in the preceding 12 months or unscheduled visit for worsening HF within 90 days, who had an ICD or CRT-D implanted with the HeartLogic^TM^ feature. As anticipated, diuretics were the most commonly adjusted medications following an alert (585 observations total), and there was a significant decline in NT-proBNP values at the 12-month follow-up visit when compared to those at enrollment (743 pg/mL [336; 1681] vs. 1316 pg/mL [664; 2856], respectively) [23]. Similarly, in the real-world observational RE-HEART study, there was a significant association between alerts and HF events, including hospitalization, mortality, and minor decompensations over a 16-month follow-up period [49]. Additionally, the NT-proBNP levels were significantly higher in patients during an active alert state (7378 vs. 1210 pg/mL; *p* < 0.001) [50].

While the HeartLogic^TM^ algorithm can be utilized to predict HF events, some challenges must also be mentioned and acknowledged. While artificial intelligence (AI) is excellent in deriving information from objective data, it cannot seamlessly integrate some critically important variables when considering the clinical condition of a patient. These include subjective symptom severity and change in functional status, among others. For example, this gap may lead to false positive alerts, as observed in a study performing real-world analysis of the HeartLogic^TM^ system [51]. This study included 107 patients with chronic HF, and approximately 26% of the device alerts were found to be false positives [51]. Moreover, the study presented an established protocol to evaluate alerts further and to guide clinical decision making by a designated HF nurse contacting the patients to determine the presence of signs and symptoms of worsening congestion. This extra step in the expert data interpretation is important in setting a relatively high false positive alert rate, as medication changes driven solely by device alerts could have had undesirable clinical consequences. However, implementing such an approach is time-consuming, costly, and likely prohibitive for most hospital systems, especially if a significant number of patients are being monitored. In addition, adjustments in the algorithm may be necessary as notifications continued in their first iteration despite improving HeartLogic^TM^ values and the lack of patient symptoms. Further studies are needed to solidify the clinical utility of HeartLogic^TM^ alert-based patient management.

### 3.3 Noninvasive Hemodynamic Monitoring Platforms

**Fig. 1.**
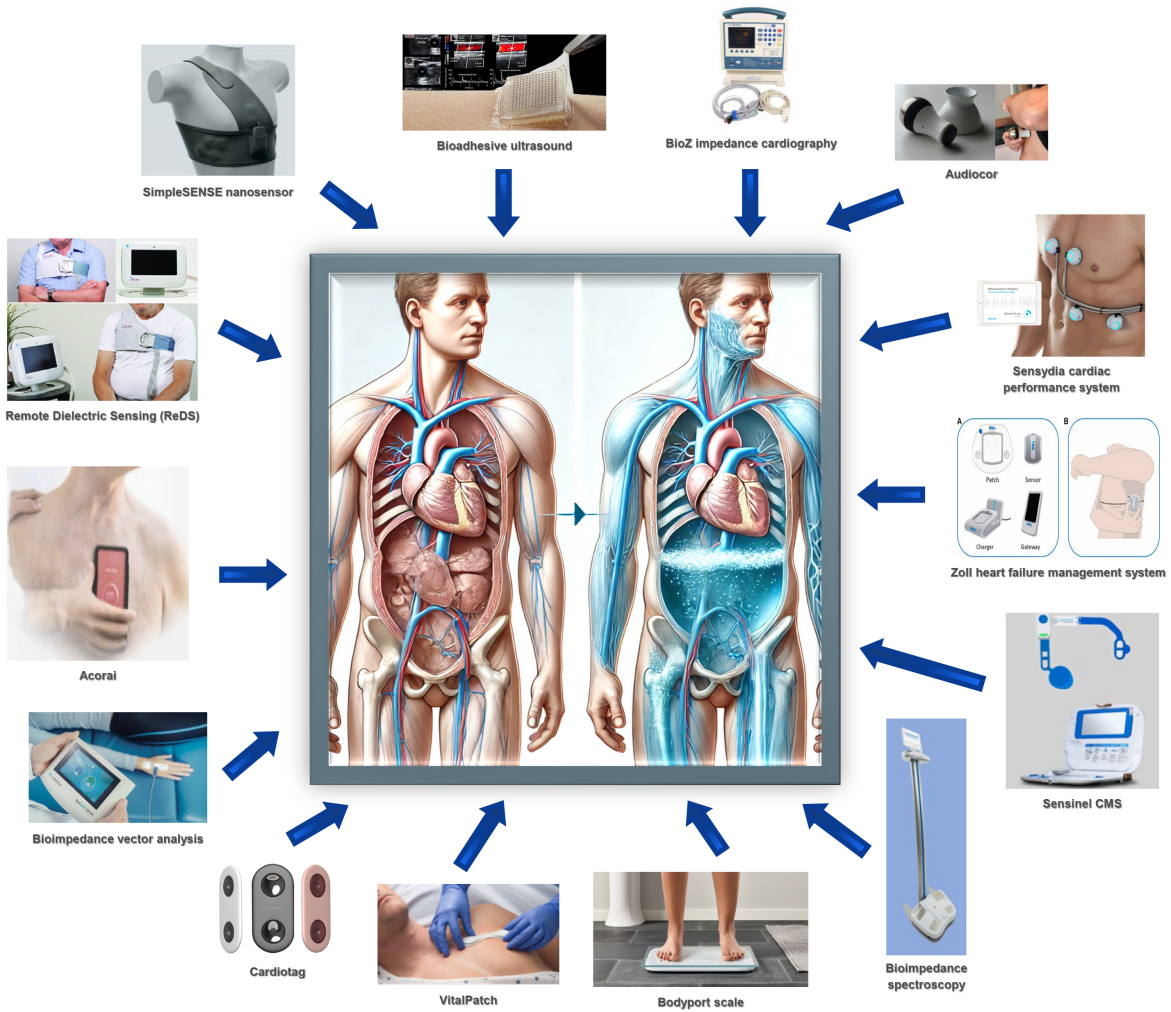
**Summary of noninvasive hemodynamic monitoring platforms**.

The clinical utility of PA pressure-based monitoring and HF management was demonstrated in the various clinical trials briefly discussed above. However, while these implanted devices reduced readmission risk and healthcare burden alongside improving mortality and quality of life of those with HF independent of left ventricular function, an invasive procedure is necessary to deploy these sensors. Meanwhile, not everyone is prepared to undergo such a procedure for various reasons, while others may be suboptimal candidates. Furthermore, sensor implantation requires specialized provider/staff training and equipment. Therefore, there is a growing need to develop noninvasive remote HF management platforms that are reliable in predicting clinical deterioration, easy to apply/remove, highly automated, that function largely independently of patient interventions, relatively cheap, and easy to scale. Several such systems have been developed over the past years that utilize a variety of technological approaches. Select noninvasive hemodynamic monitoring platforms are summarized in Figure 1 (Fig. 1).

#### 3.3.1 Venous Excess Ultrasound (VexUS)

Ultrasonography is a fast, safe, and relatively inexpensive imaging modality that can be performed at the bedside to establish volume status noninvasively. Ultrasonography may be employed to evaluate the lung parenchyma for B-lines or to measure the IVC diameter. However, image acquisition is user-dependent, making serial comparisons challenging. The VexUS is a noninvasive, reproducible, semi-quantitative platform that was developed to improve estimations of the degree of venous congestion [52]. The grading system employed in ultrasonography is based on the IVC diameter and flow patterns across the hepatic, portal, and renal venous systems [52], with higher grades corresponding to increasingly severe congestion. A recently published study by Anastasiou *et al*. [53] evaluated the utility of the VexUS score in patients requiring hospital admission for acute HF exacerbation. Of the 290 enrolled individuals, 39% had the highest VexUS score of 3, which was significantly associated with in-hospital mortality [53]. While the methodology shows promise in the acute setting, further studies are needed to evaluate its potential utility in managing patients with chronic HF in the outpatient setting.

#### 3.3.2 Bioadhesive Ultrasound (BA US)

Subsequently, BA US has been developed to potentially overcome some inherent challenges associated with ultrasonography, such as image acquisition and dynamic physiological changes within the organ of interest over time. This system consists of a thin, stiff ultrasound probe with high-density piezoelectric elements attached to the skin over the area of interest using a soft yet durable anti-drying hydrogel elastomer [54]. This probe may remain in place for 48 hours, enabling continuous high-resolution image acquisition and, thus, detecting and documenting dynamic changes in the tissues beneath. Currently, data on the use of BA US in patients with HF remain limited; however, BA US may have a future role in the early detection of worsening congestion, disease progression, and assessing response to diuretics.

#### 3.3.3 Remote Dielectric Sensing (ReDS) Platform

The ReDS (Sensible Medical Innovations Ltd., Raleigh, NC, USA) platform is a device that monitors changes in the dielectric property of the lung over time to estimate pulmonary fluid content [55]. Designed as a vest, the ReDS platform consists of a transmitter positioned over the back of the patient and a sensor placed over the anterior chest to detect the low-power radiofrequency (RF) signals after these traverse the thorax [55]. The RF signal amplitude and propagation velocity are affected by the fluid content in the lung tissue through which the signal travels, allowing the early detection of pulmonary congestion, well before symptoms develop. The ReDS platform is a versatile system that may be used in the patient’s home, clinic, or hospital setting [56]. Noninvasive ReDS values show a strong correlation with both computed tomography (CT) evaluations of lung fluid content and invasively measured PCWP, with a sensitivity and specificity of 90.7% and 77.1%, respectively, to predict a PCWP ≥18 mmHg [57]. A ReDS value <34% exhibits a negative predictive value of 94.9%. In one retrospective cohort study of 220 patients seen in clinic within 10 days from hospital discharge for HF decompensation, the ReDS platform was used to assess volume status and guide decongestive therapy, exhibiting a significantly lower rate of 30-day cardiovascular rehospitalizations with a hazard ratio (HR) of 0.21 [0.05; 0.89]; *p* = 0.04 [58]. Several other studies have also demonstrated the potential clinical benefits of ReDS-based volume management in decreasing readmission risk after HF-related hospitalizations, improving quality of life, and reducing societal healthcare burdens [59, 60].

#### 3.3.4 Zoll Heart Failure Management System (HFMS)

The Zoll HFMS (Zoll Medical, Chelmsford, MA, USA) is an FDA-approved, patch-based wireless system that uses RF technology to sense subtle increases in pulmonary fluid levels, serving as an early indicator for HF decompensation. This management system is worn 24 hours a day along the mid-axillary line and works by continuously detecting changes in returning radar signal strength and path delay due to interstitial edema. Ultimately, the platform analyzes the dynamic signals to establish the thoracic fluid index [61]. This is integrated with additional physiological variables such as the heart rate, an electrocardiogram (ECG), respiratory patterns, patient activity, and body posture. The data are automatically transmitted to the attention of the care team, thus allowing for individualized, proactive interventions. The BMAD trial revealed a significant reduction in recurrent HF hospitalizations at 90 days (HR 0.62; *p* = 0.03) and a 38% reduction in the 90-day composite outcome of recurrent HF hospitalizations, emergency department visits, or death (HR 0.62; *p* = 0.02) when using HFMS data to guide HF management [61]. Furthermore, a significantly higher improvement was observed in patient-reported quality of life as measured by the Kansas City Cardiomyopathy Questionnaire-12 questionnaire in the treatment arm compared to the control group (12 points difference on average; *p* = 0.004) [60]. However, there are certain limitations to the trial that are important to note: (1) the lack of randomization as it was a concurrent-control trial; (2) the slightly different enrollment periods for the study arms; (3) the potential lack of compliance with wearing the device or inconsistent time frame from receiving the thoracic fluid index data to performing an intervention. These may lead to underestimating the true potential of this novel platform [62].

#### 3.3.5 BioZ Impedance Cardiography (ICG)

Noninvasive ICG (CardioDynamics, San Diego, CA, USA) assesses CO, thoracic fluid content, and blood flow velocity within the aorta by detecting changes in blood conductivity throughout the cardiac cycle. Multiple patches are placed along the neck and thorax to detect instantaneous changes in electrical resistance (impedance) [63]. The initial study enrolled 212 stable HF patients with recent decompensation to undergo blinded testing using the BioZ ICG monitor. The data demonstrated that a composite score, consisting of three ICG parameters (velocity index, thoracic fluid content index, and left ventricular ejection time), which positively correlated with the risk of decompensation and served as a strong predictor for future HF events (*p* = 0.0002) [64]. However, the subsequent prospective sub-study of the ESCAPE trial (BioImpedance CardioGraphy trial) only detected a modest correlation between the ICG derived from the BioZ monitor and the invasively measured CO in hospitalized patients with advanced HF [65]. Furthermore, the study revealed that the estimated thoracic fluid content was not a reliable measure of PCWP and that ICG variables considered alone or combined were not accurate prognostic predictors within 6 months after hospitalization [65]. Hence, further research is needed to improve understanding and refine the utility of BioZ ICG as a remote management tool for patients with HF.

#### 3.3.6 Bioimpedance Spectroscopy (BIS)

BIS is a system that uses surface electrodes, placed on predefined areas of the body, to generate electrical currents with different frequencies [66]. Moreover, the sensor detects specific impedance levels based on the conductivity and dielectric properties of the target tissue [66]. By analyzing these data, BIS can estimate the total body, extracellular, and intracellular water content and body fat mass [67]. BIS testing was utilized in a recent clinical trial to measure and monitor the degree of fluid accumulation in 67 participants (42 patients admitted for decompensated HF and 25 healthy controls) [68]. This revealed that extracellular resistance across various vectors was significantly lower in those with decompensation: “whole-body” (*p* < 0.001), “foot-to-foot” (*p* = 0.03), “hand-to-hand” (*p* < 0.001), and “transthoracic” (*p* = 0.014). As patients improved with therapy, their BIS values changed and became similar to the control group [68]. A clinical trial evaluating the efficacy and safety of home-based BIS monitoring is currently underway with results expected in the near future [69].

#### 3.3.7 Bioimpedance Vector Analysis (BIVA)

BIVA is a noninvasive technique that measures human body composition, specifically regarding nutritional and hydration status. BIVA is useful in predicting hospital length of stay in addition to short- and long-term survival when combined with other established prognostic parameters obtained routinely in patients with HF [70, 71, 72]. In a prospective single-center study that evaluated BIVA parameters for patients admitted with HF and underlying congenital heart disease, the edema index (EI) was found to correlate closely with higher serum NT-proBNP levels (r = 0.51; *p* < 0.001). In addition, the EI significantly differed between patients with NYHA Class III–IV (0.398 ± 0.011) and I–II (0.384 ± 0.017; *p* < 0.001) HF symptoms [70]. A Kaplan–Meier analysis revealed that a discharge EI >0.386 was associated with a significantly increased risk of future HF-related admissions (HR = 4.15 [1.70–11.58]; *p* < 0.001) [70].

The bioelectrical phase angle (BPA) is a bioelectrical impedance measurement that corresponds with decreased cell integrity, and it is another important BIVA-derived parameter recently found to be a potential biomarker in HF patients [71]. A BPA <4.2° can independently predict mortality in patients with HF (relative risk: 3.08) [72]. Conversely, a BPA ≥5.7° corresponded with improved survival as confirmed by a Kaplan–Meier analysis [73].

#### 3.3.8 Bodyport Scale

The Bodyport scale (Bodyport Inc., San Francisco, CA, USA) is a versatile, noninvasive platform that identifies HF decompensation early using changes in various parameters obtained while simply stepping on a cardiac scale. The scale measures the body weight and detects certain signals through the feet in approximately 30 seconds; moreover, it uses advanced AI-based algorithms to translate these readings into numerous biomarkers. Among others, these include heart rate, heart rhythm using single-lead ECG, and estimated fluid content determined by multifrequency vectors to analyze impedance signals. Ultimately, an integrated parameter termed heart function index (HFI) is reported directly to the user. In addition, the scale transmits the information wirelessly to a web-based platform, which generates an electronic dashboard for providers to review. Following the interpretation, patients are notified of any potentially necessary treatment modifications. Although the platform has gained FDA approval, it is still under testing in the prospective, multicenter, observational SCALE-HF-1 study designed to assess the performance of HFI in predicting worsening HF events [74]. Preliminary results demonstrated a moderate sensitivity with HFI predicting 48 of 69 HF events correctly (70%), and there were 38% fewer false alerts when compared to traditional weight-based monitoring systems [74]. The improved sensitivity and lower false positive alert rates help improve workflow efficiency, avoid unnecessary testing and interventions, and minimize patient anxiety. Furthermore, the platform’s congestion index alerted providers on average 14 days before a potential event, allowing sufficient time to alter the HF management plan and prevent potential hospitalization. However, there was a 30% false negative rate [74]. These results highlight the importance of cautious AI-derived result interpretation at this time and the need for ongoing monitoring and expert evaluation. Another limitation of the trial is that only three FDA-approved scale-derived biomarkers (weight gain, change in impedance magnitude, and absolute impedance magnitude) were used to derive HFI and formulate the alert algorithm. Subsequent studies should focus on fine-tuning this process and potentially including further biomarkers, such as ballistocardiographs, as planned in the SCALE-HF2 trial [75].

#### 3.3.9 Audicor Remote Patient Management System

The Audicor remote patient management system (Inovise Medical, Beaverton, OR, USA) is a portable, hand-held device that uses cardiac acoustic biomarkers (CABs) and ECG tracing to assess for worsening congestion associated with HF decompensation events. Machine learning algorithms are employed to assist with data integration and interpretation. One of the key CABs utilized by the platform is the electromechanical activation time (EMAT), which refers to the time interval between the QRS complex onset and the first heart sound (S1). EMAT prolongation has been associated with left ventricular (LV) dysfunction. In a recent randomized, controlled, single-blinded trial, patients admitted with acute HF exacerbation were randomly assigned to one of two groups before discharge: (1) Audicor CAB-guided HF interventions; (2) traditional, symptom-based outpatient management strategy [76]. The study found that the group with CAB-guided management experienced a significantly lower rehospitalization rate compared to the controls after a follow-up period of 238 ± 141 days (43 vs. 61 events; *p* < 0.001) [76]. Further studies are ongoing for this novel platform.

#### 3.3.10 VitalPatch

VitalPatch (VitalConnect, San Jose, CA, USA) is a wearable biosensor attached to the chest wall using adhesives. This sensor continuously collects and integrates numerous physiological parameters, such as ECG tracing, heart rate, respiratory rate, patient posture, and physical activity. These variables are uploaded to a cloud-based system (PhysIQ), and a machine learning algorithm calculates an early warning score (EWS). The LINK-HF study was a multicenter, observational study that examined the clinical performance of this personalized analytical platform using a continuous data stream in predicting HF rehospitalizations in 100 subjects with Class II–IV symptoms. VitalPatch was able to detect the precursors for admission with a sensitivity of 76–88% and a specificity of 85%, and with a median lead time of 6.5 (4.2–13.7) days [77]. However, major limitations of the study included a predominantly male population (98%), the omission of five events due to compliance issues, and the absence of a separate group to validate the algorithm. These limitations prompted the development of the follow-up LINK-HF2 trial.

The recently completed LINK-HF2 multicenter study (NCT04502563) included veterans with Class II–IV HF symptoms and consisted of two phases [78]: (1) a vanguard phase that enrolled 27 patients, aimed at making protocol adjustments to improve the system’s usability for both clinicians and participants. This phase included adjustments to the alert algorithm aiming to minimize the false alert rate and patient and clinician education; (2) multicenter, randomized phase. The ultimate goal of the LINK-HF2 trial was to assess whether this technology can effectively identify patients at risk for worsening HF and to facilitate medication adjustments, thereby preventing the need for hospital admission. Results are pending and were not published during the preparation of this review.

#### 3.3.11 CardioTag

CardioTag (Cardiosense, Chicago, IL, USA) is a small (approximately the size of a business card), portable, noninvasive, multisensor wearable cardiac monitoring device designed to identify markers of cardiac disease in the presymptomatic period. The device operates by collecting and integrating a variety of parameters, such as the seismocardiogram (SCG), ECG, and photoplethysmogram (PPG), with the primary intent to estimate a range of hemodynamic parameters. Patients must only place their fingers over the sensors for 30 seconds to gather the necessary data. Multiple studies have demonstrated a strong correlation between the changes in hemodynamic parameters (PCWP, mean arterial pressure, stroke volume, CO) as estimated by CardioTag and the gold-standard measurement [79, 80, 81, 82]. The FDA has granted this novel platform the breakthrough device designation for its potential to identify patients at risk for HF decompensation, specifically targeting individuals with NYHA Class III and IV symptoms.

#### 3.3.12 Acorai Heart Monitor

The Acorai Heart Monitor (Acorai, Helsingborg, Sweden) is a multisensor handheld device that employs the SAVE sensor system, combining seismocardiography, phonocardiography, photoplethysmography, and ECG. The Acorai Heart Monitor was developed to produce accurate, absolute, and actionable information on various hemodynamic parameters that can be estimated serially. The system obtains the necessary signals following placement over the patient’s chest for approximately 2 minutes while supine. Using machine learning technology, the Acorai Heart Monitor can estimate the right atrial pressure, PA pressure, PCWP, and CO. In an observational study that enrolled 281 subjects, investigators found a good correlation between the mean pulmonary artery pressure (mPAP) as estimated by the Acorai Heart Monitor and by the standard of care invasive RHC (*p* = 0.75; r^2^ = 0.55; mean difference of measurement: 0.78 mmHg) [83]. To further establish the clinical utility of the platform, the results were compared with the ESCAPE bedside evaluation data. In this randomized, controlled trial, severe congestion was defined as a PCWP >22 mmHg. The areas under the curve were 0.74, 0.63, and 0.55 for the Acorai Heart Monitor, cardiologist-led evaluation, and biomarker levels, respectively [84]. The investigational device exhibited a sensitivity of 95% and a specificity of 31%, making it particularly well suited for screening [84].

#### 3.3.13 Sensinel Cardiopulmonary Management (CPM) System

The Sensinel CPM (Analog Devices, Wilmington, MA, USA) system is a novel, wearable device developed for the early detection of cardiopulmonary conditions in the home setting. The Sensinel CPM system consists of a wearable and a base station. The system integrates input from many sensors that capture various physiological parameters, such as body temperature, heart sounds, heart rate, single-lead ECG, relative tidal volume, respiratory rate, thoracic impedance, and body posture. Information is submitted to a cloud-based platform where proprietary algorithms are employed to detect trends and identify worsening cardiopulmonary conditions early. The device is simple to operate and is self-applied by the patient to their chest wall for approximately 3–5 minutes daily. The CONGEST-HF trial evaluated the effectiveness of the CPM system in 66 hospitalized patients admitted for volume overload requiring decongestion [85]. Investigators found that the platform could accurately detect changes associated with fluid removal and weight loss [85]. In patients undergoing hemodialysis, the fluid volume removed during a session correlated well with changes in thoracic impedance as detected by the CPM (rₛₚ = 0.49; *p* = 0.024) [85]. However, no strong correlation was observed between the invasively measured and platform-estimated PCWP values [85]. Further, large-scale studies are planned to evaluate whether the system can reliably detect congestion in the patient’s home setting and if it provides enough lead time to implement changes in HF management so that hospitalizations and ER visits may be avoided or reduced.

#### 3.3.14 SimpleSENSE Nanosensor

The SimpleSENSE (Nanowear, Brooklyn, NY, USA) nanosensor is currently the only noninvasive, cloth-based, FDA-cleared nanosensor. Further, designed as a wearable monitoring undergarment, this nanosensor combines impedance cardiography, thoracic impedance, phonocardiography, ECG, respiratory rate, and physical activity monitoring to generate a score that can be used to predict impending HF exacerbation [86]. The NANOSENSE study is an ongoing multicenter, prospective feasibility trial designed to enroll 500 patients over two years who had a recent HF hospitalization (NCT03719079). The primary goal of the trial is to develop and validate an algorithm for predicting HF progression. Participants wear the SimpleSENSE garment for 12 hours daily, predominantly during sleep, to provide continuous monitoring.

#### 3.3.15 Sensydia Cardiac Performance System (CPS)

The Sensydia CPS (Los Angeles, CA, USA) is a novel, noninvasive portable, point-of-care device under development that uses cardiac acoustic and ECG signals in conjunction with machine learning algorithms to estimate LVEF and a range of hemodynamic parameters, including mPAP and CO. Multiple studies have been conducted to evaluate and validate the accuracy of the system. In one of these trials that enrolled 38 patients, the Bland–Altman analysis revealed a bias of –0.075 and limits of agreement between CPS-CO and RHC thermodilution CO of [–1.78, 1.63] L/min [87]. The CPS also demonstrated an ability to identify patients with an LVEF below 40%, achieving an area under the curve of 0.93 [88]. In 18 patients with HF and preserved ejection fraction (HFpEF), the Bland–Altman calculated bias and limits of agreement between the E/A ratio obtained from phonocardiogram-based and Doppler echocardiogram-derived measurements were 0.00 ± 1.28 [89]. In addition, a clinical trial (NCT06149143) was recently completed to optimize the ability of the system to predict CO, PA pressure, and PCWP. These results were pending publication at the time of this review.

## 4. Limitations and Challenges of Noninvasive Hemodynamic Monitoring Platforms

The simple fact that an invasive procedure is not required to deploy a sensor permanently means that wearable technology has an advantage over implantable platforms and may significantly expand the potential target population. However, these rely on a combination of surrogate markers to estimate congestion. Thus, their accuracy remains a significant concern compared to invasive hemodynamic monitoring, particularly the most widely available PA pressure sensors. Presently, most devices present a limited dataset collected in relatively small cohorts, thereby restricting their generalizability and emphasizing the need for larger trials in the future. Similar to implanted sensors, patient compliance with measurements remains of paramount importance.

## 5. Telemedicine and Device Monitoring in Patients With HFpEF

Although more than half of the patients with HF are considered to have HFpEF based on the normal LV function, diagnosis and management remain a clinical challenge primarily due to the heterogeneous nature of the disease. The number of medications and device therapies available for this cohort is much more limited than those with HF and reduced ejection fraction (HFrEF). In addition to GDMT titration, treating comorbidities, weight loss, blood pressure control, exercise training, and symptom control remain the priorities. These correlate closely with volume status management, especially considering that congestion and elevated PA pressures are the primary drivers responsible for the cardinal signs and symptoms of HF. As such, remote patient management may be particularly critical in this growing group of patients. Recognizing this fact, several clinical trials with implantable sensors have subsequently enrolled patients with HFpEF and HFrEF to perform prespecified subgroup analyses. For example, these include the HOMEOSTASIS, CHAMPION, GUIDE-HF, VECTOR-HF, SIRONA, PROACTIVE-HF, and FUTURE-HF trials [13, 17, 18, 21, 28, 29, 33]. Some of these have demonstrated a clear benefit in the remote management of HF in patients with HFpEF, often exceeding the advantage documented in those with reduced ejection fraction. Notably, data using CIED sensors in the HFpEF population is scarce because these are guideline indications for those with an LVEF at or below 35%.

Focusing on noninvasive platforms, the BMAD, LINK-HF-1, LINK-HF-2, and SCALE-HF-1 studies enrolled patients independent of their LVEF. These trials have demonstrated similar primary outcomes as those with HFpEF and HFrEF, effectively reducing HF-related hospital admissions across the HF spectrum [61, 74, 77, 78]. Most of the recently completed and currently active clinical trials in the field enroll patients with HFpEF, and future studies will likely enrich their participants for this subgroup of patients. Managing congestion efficiently is anticipated to derive a significant clinical benefit for this cohort, reducing symptom burden and potentially improving survival.

## 6. From Trials to Clinical Practice: Implementation and Cost-Benefit of Invasive and Noninvasive Monitoring Technologies for Heart Failure

While monitoring platforms for patients with HF offer promising opportunities for early congestion detection, the integration of these platforms into an existing clinical workflow often poses a challenge. CardioMEMs^TM^ is the most established among the currently FDA-approved systems, with real-world data demonstrating its clinical utility and cost-effectiveness [34, 36, 90]. The use of CardioMEMs^TM^ has been associated with an incremental cost-effectiveness ratio (ICER) of USD 82,301 in patients with HFrEF and USD 47,768 with HFpEF per quality-adjusted life year (QALY) [91]. More recently developed invasive hemodynamic monitoring devices, such as the Cordella^TM^ and V-LAP, are in the early stages of clinical validation with only limited real-world data as of this publication. Similarly, evidence supporting the cost-effectiveness of the HeartLogic^TM^ algorithm and the noninvasive monitoring platforms remains limited [92]. From the standpoint of the healthcare system, some challenges need to be mentioned. One relates to the hospital staffing model, as monitoring continuous data inflow from various remote platforms requires specialized, dedicated, highly trained nurses and providers. These trained personnel need to be familiar with the patients and access the most up-to-date management plan and their laboratory information. In addition, communication must be efficient and timely. Other than larger health care systems, dedicating one or multiple full-time equivalents (FTEs) to perform these duties solely may be challenging, especially with limited insurance reimbursement. Using the “manage by exception” strategy is certainly helpful in reducing the clinical burden, yet data review and regular documentation of these activities are still mandatory. Another challenge is related to electronic medical record (EMR) integration, as providers are often tasked with monitoring several web-based platforms simultaneously. This also limits data availability to the select few with approved access. EMR integration has to be completed for each monitoring platform individually; it is costly and time-consuming, often taking several months or more to complete based on local information technology availability. However, this is a critical step to improve and facilitate the management of patients with HF.

## 7. Future Directions and Emerging Areas of Investigation

As telemedicine and remote patient management continue to grow, several key areas remain to be explored or improved in the future. While prospective trials have demonstrated better outcomes when using select monitoring platforms, the real-life effectiveness of most systems remains uncertain. Indeed, questions regarding patient adherence with daily data submission and provider engagement in monitoring outside trial boundaries remain. Moreover, the need for improvements in the healthcare system infrastructure may delay implementation at some sites. Future studies should focus on real-world implementation, pragmatic trials, and assessing how best to integrate remote management systems into hospital workflows in large academic centers and small, rural, community-based hospitals.

Future platforms are also anticipated to focus on automation. Systems that do not mandate external intervention to perform a measurement and to submit data are more likely to be successful in improving adherence and clinical outcomes. Providing direct, patient-facing feedback will be important as many engaged individuals with HF are interested in “knowing their numbers” and are much more likely to make lifestyle changes if they are aware of their objective congestion assessment. As a subsequent step, individualized management protocols may be established that patients can follow based on their remote management data.

## 8. Conclusions

The incidence and prevalence of HF continue to grow, and reducing hospitalizations while improving the quality and quantity of life of the affected population is paramount. A wide range of implantable and wearable platforms have been developed over the past decades, designed to monitor various cardiac and extracardiac parameters and aiming to detect early signs of HF exacerbation in real time. These devices are safe, accurate, and can potentially improve clinical outcomes in this population, especially with the advent and implementation of AI and machine learning algorithms. However, these devices vary in design, the monitored parameters, analysis strategy, and data submission frequency. Today, all platforms require clinician oversight, even when utilizing a “manage-by-exception” strategy, and clear protocols need to be established on how to act upon the information received. Integration into various existing hospital EMR systems remains scarce. Monitoring multiple platforms in parallel is a time-consuming challenge for medical professionals and may limit the widespread adaptation, at least temporarily, of these emerging technologies. Further research is needed to identify the ideal remote management approach. Nevertheless, both invasive and newly developed noninvasive platforms seem to have the potential to achieve their stated clinical goals of improving the quality and quantity of life of patients living with HF.
